# Evaluation of serum neurofilament light chain, GFAP, and peripherin as biomarkers in hereditary transthyretin amyloidosis

**DOI:** 10.1038/s41598-026-56777-y

**Published:** 2026-06-08

**Authors:** Intissar Anan, Gabriella Johannson, Björn Pilebro, Jonas Wixner, Victoria Heldestad, Sandra Arvidsson, Malin Olsson

**Affiliations:** 1 Department of Public Health and Clinical Medicine, Umeå University, Umeå, Sweden; 2 Department of Clinical Microbiology, Umea University, Umeå, Sweden; 3 Department of Diagnostics and Intervention, Umea University, Umeå, Sweden

**Keywords:** Transthyretin, Amyloidosis, Neurofilament light chain, Neuropathy, Biomarker, Biomarkers, Diseases, Medical research, Neurology, Neuroscience

## Abstract

Early diagnosis and accurate monitoring of disease progression are crucial for timely therapeutic intervention in hereditary transthyretin amyloidosis (ATTRv). Neurofilament light chain (NfL) has emerged as a sensitive biomarker of neuroaxonal injury across neurodegenerative disorders. This study aimed to investigate serum levels of NfL, glial fibrillary acidic protein (GFAP), and peripherin (PRPH) in patients with ATTRV30M amyloidosis and pre-symptomatic gene carriers, compared with controls. Serum samples from 34 ATTRV30M patients, 17 pre-symptomatic ATTRV30M carriers, and 35 controls were analysed using conventional commercially available ELISA platforms to quantify NfL, GFAP, and PRPH concentrations. Serum NfL (sNfL) levels were significantly elevated in ATTRV30M patients compared with controls (threefold, *p* = 0.0005) and were 1.6-fold higher than in pre-symptomatic ATTRV30M carriers. No significant differences were observed between pre-symptomatic carriers and controls. sNfL concentrations were higher in patients with polyneuropathy disability (PND) score > I compared with PND I (*p* = 0.0007). Serum GFAP and PRPH levels did not differ significantly among the study groups. Serum NfL represents a promising non-invasive biomarker for assessment of early symptomatic disease and monitoring of disease progression in ATTRV30M amyloidosis. In contrast, sGFAP and sPRPH appear to have limited diagnostic utility in this context.

## Introduction

Transthyretin (ATTR) amyloidosis is a rare multisystemic disorder caused by the extracellular deposition of misfolded transthyretin (TTR) protein in various organs and tissues^1,2^. Two main types are recognised: hereditary (ATTRv) and wild-type (ATTRwt) transthyretin amyloidosis^2^. ATTRv amyloidosis is caused by genetic variants in the TTR gene, leading to more amyloidogenic TTR protein. ATTRv can occur at any adult age and often presents with peripheral and autonomic neuropathy as well as cardiac manifestations^3^. More than 200 variants in the TTR gene have been described (Human Gene Mutation Database: www.hgmd.cf.ac.uk), and clinical expression varies according to genotype and other modifying factors^4^. In contrast, ATTRwt amyloidosis primarily affects elderly individuals resulting from aggregation of non-genetically altered TTR, typically in the heart^5,6^.

TTR is predominantly synthesised in the liver and serves as a plasma transport protein for thyroxine (T4) and retinol-binding protein (vitamin A complex). In ATTRv amyloidosis, genetic variants destabilise the native TTR tetramer, causing dissociation into monomers that misfold and aggregate into amyloid fibrils^7^ Two fibril types have been identified: type A, consisting of a mixture of fragmented and full-length TTR, and type B, composed solely of full-length TTR^8^. Type A fibrils are typically associated with later onset and mixed neuropathic and cardiac involvement, whereas type B fibrils correspond to earlier onset and predominantly neuropathic symptoms. Notably, the Swedish ATTRV30M population exhibits both fibril types, whereas non-V30M variants in Sweden tend to display only type A fibrils^9,10^.

The degree of polyneuropathy varies between TTR variants, and early diagnosis is essential to prevent irreversible nerve damage^11^. Combined nerve conduction studies (NCS) and electromyography (EMG) remain the mainstay for assessing large myelinated fibres^12^. These techniques are time-consuming, resource-intensive, and often uncomfortable for patients^13^, furthermore, their sensitivity in early disease stages are limited. There is therefore a growing need for novel, minimally invasive biomarkers to assist early diagnosis and disease monitoring.

Among candidate biomarkers of peripheral nerve injury, neurofilament light chain (NfL) has emerged as particularly promising^14,15^. In addition, glial fibrillary acidic protein (GFAP)—an intermediate filament protein released from astrocytes during reactive gliosis—serves as a marker of central nervous system injury. Similarly, peripherin (PRPH), a type-III intermediate filament expressed in peripheral neurons, may reflect peripheral axonal degeneration. Together, these three structural proteins capture complementary aspects of nervous system pathology.

Among them, neurofilaments are the principal structural components of the axonal cytoskeleton, comprising three subunits: neurofilament light (NfL), neurofilament medium, and neurofilament heavy chains^16,17^. Neurofilaments are released into the extracellular space following axonal injury and subsequently enter the cerebrospinal fluid and bloodstream.

The present study aimed to evaluate serum concentrations of NfL, GFAP, and PRPH in Swedish patients with ATTRV30M amyloidosis, pre-symptomatic ATTRV30M carriers, and matched controls. We hypothesised that both patients and carriers would exhibit elevated levels of these biomarkers compared with controls, reflecting underlying neuroaxonal injury.

## Materials and methods

### Study population

The study cohort consisted of 86 participants, 51 of these had a confirmed pathogenic TTR Val30Met (p.Val50Met, ATTRV30M) variant and included both symptomatic and pre-symptomatic. The remaining 35 participants in the study cohort were matched controls. The diagnosis of ATTRv amyloidosis was established according to Swedish national clinical practice and based on TTR gene sequencing, histopathological examination. Briefly, the TTR variant was identified by sequencing all four exons of the TTR gene using conventional Sanger sequencing (done by Clinical Genetics at Umea University hospital). Histopathological analysis of subcutaneous adipose tissue biopsies was performed using Congo red staining and polarised light microscopy to confirm the presence of amyloid. Amyloid precursor protein and fibril type were determined by Western blot analysis of abdominal subcutaneous adipose tissue, as previously described^18^. Cardiac amyloidosis was assessed and supported diagnostically by 99mTc-diphosphono-1,2-propanodicarboxylic acid (DPD) scintigraphy. DPD uptake corresponding to Perugini grade I–III was considered diagnostic for cardiac amyloid involvement and was used as part of the overall diagnostic evaluation. The DNA of controls was genotyped to confirm the absence of ATTRV30M variant, and none of the controls had a history of polyneuropathy, known neurological disease, or other systemic disorders associated with peripheral nerve injury.

Clinical data were extracted from the medical records of Umeå University Hospital, including initial symptoms, clinical manifestations, amyloid fibril type, and relevant laboratory findings. All serum samples were collected prior to initiation of disease-modifying treatment. The severity of polyneuropathy was graded according to functional capacity using the Polyneuropathy Disability (PND) score: PND I (sensory symptoms with preserved walking ability), PND II (impaired walking without aid), PND IIIa (requires one walking aid), PND IIIb (requires two walking aids), and PND IV (wheelchair- or bed-bound).

### Biomarker measurements

Serum concentrations of NfL, GFAP, and PRPH were measured using enzyme-linked immunosorbent assay (ELISA). Blood was collected in serum tubes without gel separators, allowed to clot, and centrifuged at 1,500 × g for 15 min at room temperature. Serum aliquots were stored at − 80 °C until analysis.

Serum NfL was quantified using a commercial ELISA kit (Cat No 20-8002; UmanDiagnostics AB, Umeå, Sweden). GFAP (Cat No EEL079; Invitrogen, Thermo Fisher Scientific Inc., USA) and PRPH (Cat.No BSKH64124; Bioss Inc., USA) concentrations were determined using manufacturer-specified ELISA kits. All assays were performed in duplicate according to the manufacturers’ instructions.

Briefly, blanks, standards, and appropriately diluted serum samples were pipetted into coated 96-well plates and incubated at the recommended temperature according to respective ELISA kit. After washing, conjugated detection antibodies were added and incubated, followed by substrate solution. The reaction was stopped with acid stop solution, and absorbance was measured at 450 nm using microplate reader SkyHigh (Thermo Scientific Multiskan, USA). The serum concentrations of the biomarkers were automatically calculated using SkanIt software (Thermo Scientific, USA). The accepted coefficient of variance (CV%) between sample duplicates was ≤ 15%. Serum samples with absorbance just below the lower Limit of Detection (LOD) but within the permitted extrapolation range were given a set LOD value.

### Statistical analysis

Statistical analyses and graphics were performed using GraphPad Prism 10.6.1 software (GraphPad Software, Boston, Massachusetts USA). Continuous variables were presented as median (25th-75th percentiles) or mean [range], and categorical variables as count (percentage).

A normal distribution test was performed on the continuous variables using the Shapiro–Wilk test and it showed significant deviations from normality. Hence, comparisons between two independent groups were performed using the non-parametric Mann-Whitney U test for two independent samples. For comparisons involving more than two groups, the non-parametric Kruskal–Wallis test was applied with Dunn’s correction for multiple testing. A p-value < 0.05 was considered statistically significant.

## Results

### Characteristics of the study population

The demographic and clinical characteristics of ATTRV30M patients, pre-symptomatic ATTRV30M carriers, and controls are summarised in Table 1.

A total of 86 participants were recruited, comprising 34 ATTRV30M patients (18 with fibril type A and/or DPD positive (grade I-III), 16 with type B or DPD negative), 17 pre-symptomatic ATTRV30M carriers, and 35 controls. The median age at sampling was 62.4 years (25th-75th percentile: 56.1–64.9 years) in patients, 55.7 years (25th-75th percentile: 48.0-65.6) in pre-symptomatic carriers, and 60.7 years (25th-75th percentile: 46.2–67.2) in controls. The non-parametric Mann-Whitney U-test showed no significant differences in age or sex distribution between groups. The number of subjects varied slightly between the different biomarker analyses due to CV% > 15%, material shortages or failed ELISA analysis.

Initial symptoms among patients were predominantly neurological (91.2%), followed by cardiac (5.9%), ocular (5.9%), and gastrointestinal (GI) manifestations (2.9%). During the course of the disease, additionally 62% of the patients developed cardiomyopathy, and 74% experienced GI manifestations. All patients except one had evidence of neuropathy at the time of the study. Nineteen patients had Polyneuropathy Disability stage I and the remaining 15 had PND stage II or III.

**Table 1:** Characteristics and biomarker concentrations of ATTRV30M patients, pre-symptomatic ATTRV30M and controls.

		ATTRV30M	ATTRV30M	Controls
		patients	pre-symptomatic carriers	
Sex (number females/tot)		17/34	8/17	15/35
Age at sampling (median years, 25th-75th percentiles)		62.4 (56.1–64.9)	55.7 (48.0-65.6)	60.7 (46.2–67.2)
Disease duration (mean years, range)		2.9 [1–15]	n/a	n/a
Fibril type (Number)	A	18	n/a	n/a
	B	16	n/a	n/a
	Untyped	0	n/a	n/a
Symptoms at disease debut (%)	Neuropathy	91.2	n/a	n/a
	Cardiomyopathy	5.9	n/a	n/a
	Other (ocular and GI)	8.8	n/a	n/a
PND score (Number)	I	19	n/a	n/a
	> I	15	n/a	n/a
Clinical manifestations (%)	Neuropathy	97.0	0	0
	Cardiomyopathy	67.6	0	0
	GI	76.5	0	0
Biomarkers pg/mL (number; median, 25th-75th percentiles)	sNfL	32; 45.3 (18.5–81.3)	14; 19.1 (12.7–35.6)	25; 14.6 (9.8–19.9)
	sGFAP	32; 647.7 (497.3-829.6)	14; 527.9 (442.5-607.3)	25; 601.3 (485.9-704.4)
	sPRPH	27; 373.7 (26.0-784.6)	11; 26.98 (26.0-567.8)	18; 338.4 (14.6–1207.0)

*ATTRV30M* Hereditary transthyretin amyloidosis, *GI* Gastrointestinal manifestations. *PND* Polyneuropathy Disability, *sNfL* Serum neurofilament light chain, *sGFAP* Serum glial fibrillary acidic protein, *sPRPH* Serum peripherin, *n/a* not applicable.

### Serum Nfl levels

**Fig. 1:**
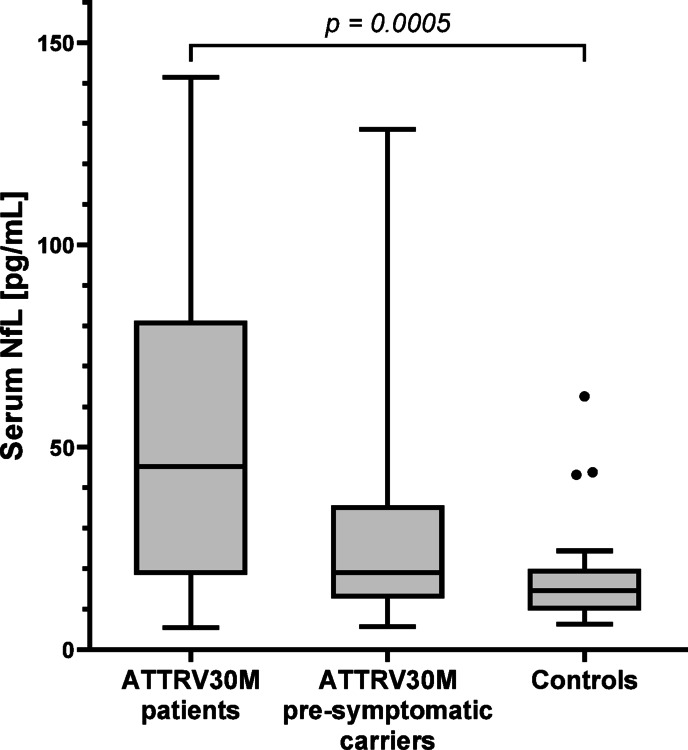
Serum neurofilament light chain (sNfL) concentrations in patients with ATTRV30M amyloidosis, pre-symptomatic ATTRV30M carriers, and matched controls. sNfL levels were significantly higher in ATTRV30M patients compared to controls.

Serum neurofilament light chain (sNfL) concentrations were significantly elevated in ATTRV30M patients compared with controls (*p* = 0.0005, Dunn’s-test adjusted), showing an approximately threefold increase. Levels were also 1.6-fold higher in patients than in pre-symptomatic ATTRV30M carriers but the Kruskal-Wallis test showed no significant difference. No significant difference was observed between pre-symptomatic carriers and controls (Fig. 1).

**Fig. 2:**
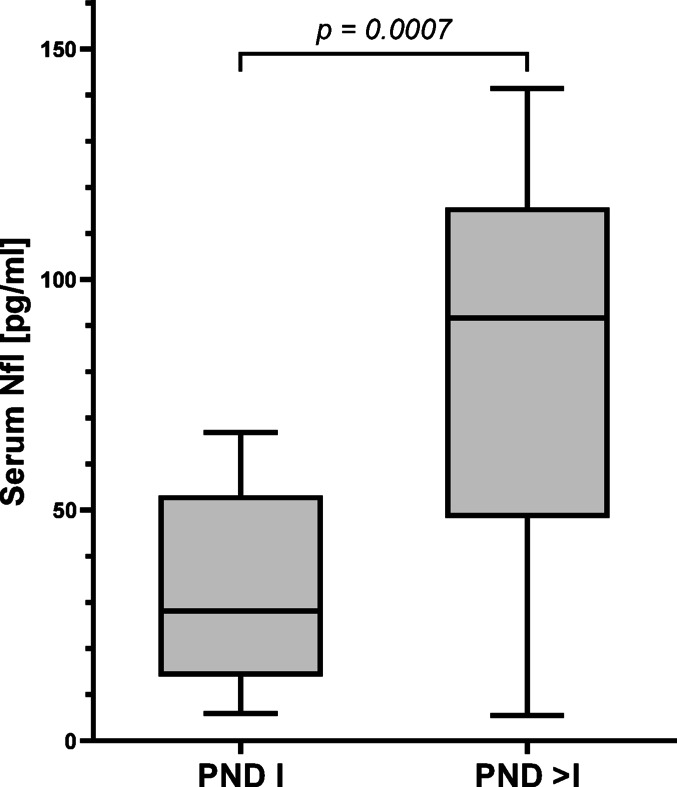
Comparison of serum neurofilament light chain (sNfL) concentrations in ATTRV30M patients with polyneuropathy disability score (PND) I and those with impaired walking capacity (PND > I), demonstrating significantly higher sNfL values in patient with PND > I compared to PND I.

There was no significant difference in sNfL levels between patients with fibril type A and those with type B. However, sNfL concentrations were markedly higher in patients with a polyneuropathy disability (PND) score > I (II and III) compared with those with PND I (*p* = 0.0007; Fig. 2). sNfL levels were also elevated in early-stage patients (PND I) compared with pre-symptomatic carriers. No significant difference was found between newly diagnosed patients and those with long-standing disease.

### Serum GFAP levels

Serum glial fibrillary acidic protein (sGFAP) concentrations did not differ significantly among ATTRV30M patients, pre-symptomatic ATTRV30M carriers, and controls. No group differences were detected between fibril type A and type B or across PND stages (I and > I). sGFAP levels were comparable between early-stage patients (PND I), and pre-symptomatic carriers, as well as between newly diagnosed patients and those with long-standing disease.

### Serum PRPH levels

Serum peripherin (sPRPH) concentrations likewise showed no significant differences among ATTRV30M patients, pre-symptomatic ATTRV30M carriers, and controls. sPRPH levels did not vary according to fibril type (A vs. B), PND stage, or disease duration. Similarly, no differences were found between early-stage patients (PND I) and pre-symptomatic carriers.

## Discussion

This study demonstrates that individuals with ATTRV30M amyloidosis exhibit markedly elevated serum neurofilament light chain (sNfL) levels compared with both controls and pre-symptomatic ATTRV30M carriers. These findings reinforce previous observations that circulating NfL reflects axonal degeneration in amyloid neuropathies^4,15^, supporting its role as a sensitive biomarker of peripheral nerve injury. In contrast, serum glial fibrillary acidic protein (sGFAP) and peripherin (sPRPH) levels did not differ significantly among groups or disease stages, indicating limited diagnostic or prognostic utility for these biomarkers in ATTRv.

### sNfL as a marker of axonal degeneration

Neurofilament light chain is a major structural protein of the axonal cytoskeleton, released into the cerebrospinal fluid and blood following axonal injury. Elevated sNfL concentrations have been reported across a wide spectrum of neurological conditions, including traumatic brain injury, stroke, multiple sclerosis, Amyotrophic lateral sclerosis, Alzheimer’s disease, and Parkinson’s disease^16–18^. Increased sNfL reflects the extent of axonal damage and correlates with disease activity and progression in both central and peripheral nervous system disorders.

In ATTRv amyloidosis, axonal degeneration is primarily driven by amyloid fibril deposition within the endoneurium and around the vasa nervorum, causing ischaemic injury and direct axonal toxicity from prefibrillar oligomers. The current study’s finding of stepwise sNfL elevation—from pre-symptomatic ATTRV30M carriers to early-stage (PND I) and advanced neuropathy (PND > I, stage II or III)—supports its potential as a dynamic marker of disease severity. No significant difference was observed between pre-symptomatic carriers and controls. The relatively large variability observed among pre-symptomatic carriers may reflect biological heterogeneity and varying proximity to symptomatic disease conversion.

These findings are in line with previous studies demonstrating elevated circulating NfL concentrations across several neurodegenerative and peripheral neuropathic disorders, supporting the concept that sNfL reflects the degree of ongoing axonal injury rather than disease-specific pathology.

Importantly, increased sNfL levels in patients with mild neuropathy compared with controls suggest that axonal degeneration begins early in the symptomatic disease course. These results align with previous longitudinal data showing that NfL rises before symptomatic onset in hereditary and inflammatory neuropathies^14,19^. Thus, sNfL may enable earlier detection of subclinical nerve injury and could facilitate monitoring of therapeutic response to disease-modifying treatments in ATTRv amyloidosis.

Future longitudinal studies are warranted to determine whether stabilisation or improvement in neuropathy corresponds to a reduction in sNfL levels. Establishing standardised assay protocols and reference ranges will also be essential for clinical implementation.

### GFAP: a CNS biomarker with limited relevance in ATTRv amyloidosis

GFAP is an intermediate filament protein primarily expressed in astrocytes, where it serves as a well-established marker of central nervous system (CNS) injury and astroglial activation. Elevated serum GFAP has been reported in various CNS disorders, including traumatic brain injury, multiple sclerosis, and Alzheimer’s disease^20–22^.

However, GFAP’s relevance in peripheral neuropathies appears limited. Astrocytes are absent in the peripheral nervous system, and although GFAP expression can increase in non-myelinating Schwann cells following injury, its systemic release is likely minimal. Consistent with previous findings in diabetic and immune-mediated neuropathies^23,24^, serum GFAP levels remained stable across all groups in our study.

However, recent evidence suggests that serum GFAP may be elevated in ATTRv amyloidosis even from pre-symptomatic stages. In a large multicentre cohort, Plantone et al. reported significantly increased sGFAP levels in both symptomatic ATTRv patients and pre-symptomatic carriers compared with controls, and proposed contributions from central nervous system, peripheral nervous system, and enteric glial involvement^25^.

The discrepancy between these findings and the present study may be explained by methodological differences, including the use of ultrasensitive digital immunoassays (Simoa) versus conventional ELISA, as well as cohort heterogeneity with respect to age, genotype distribution, and disease phenotype. Furthermore, CNS involvement such as leptomeningeal amyloid deposition—although clinically silent in many cases—may contribute to astroglial activation and GFAP release without overt neurological signs.

### Peripherin (PRPH): a peripheral neuronal filament with context-dependent sensitivity

Peripherin, a type III intermediate filament selectively expressed in peripheral neurons, has been proposed as a peripheral nerve–specific biomarker of axonal injury. Elevated peripherin levels have been reported in acute neuropathies such as Guillain–Barré syndrome (GBS), where it can distinguish GBS from other neuropathies with reasonable specificity^26^. More recently, Plantone et al.^27^ reported significantly elevated serum peripherin concentrations in both symptomatic and presymptomatic ATTRv subjects using an ultrasensitive Simoa-based assay. In contrast, our study did not demonstrate significant PRPH elevation in ATTRv amyloidosis. These discrepent findings may partly reflect methodological and cohort-related differences, including assay sensitivity, sample size, disease stage distribution, and genotype heterogeneity between studies. In addition, differences in disease kinetics may contribure to variability in circulating peripherin concentrations. ATTRv neuropathy progresses slowly and chronically, leading to gradual axonal degeneration, whereas acute conditions such as GBS are charactersied by rapid fibre disintegration and transiently high biomarker release. Consequently, chronic low-grade axonal loss in ATTRv may generate circulationg PRPH concentrations below the detection threshold of conventional ELISA platfroms. Additionally, PRPH may be cleared or degraded rapidly in circulation. Taken togheter, these findings suggest that peripherin may be more sensitive in acute peripheral nerve injuries status than in slowly progressive neuropathies.

### Integrating the biomarker profile

Collectively, our findings highlight a hierarchy of biomarker sensitivity in ATTRv amyloidosis. Serum NfL provided the most consistent and biologically plausible signal of axonal degeneration, correlating with disease severity and progression, whereas sGFAP and serum peripherin remained unchanged. This distinction underscores the importance of tissue specificity and release dynamics when interpreting circulating biomarkers.

Future studies should explore multi-analyte biomarker panels, integrating sNfL with other markers such as peripherin, myelin-related proteins (e.g., PMP22 fragments, myelin basic protein), and vascular or inflammatory mediators. Combining biochemical markers with quantitative neurophysiological, imaging, or skin biopsy data may improve staging precision and enhance sensitivity for clinical trials within ATTR amyloidosis.

### Limitations

This study has several limitations. The cross-sectional design precludes assessment of longitudinal biomarker changes over time. The sample size, particularly among pre-symptomatic carriers, was modest, which may limit statistical power and the possibility for more advanced age-adjusted multivariable analyses. In addition, no non-hereditary ATTR or other neuropathic comparison cohort was included, limiting conclusions regarding disease specificity of the observed biomarker changes. Additionally, biomarker measurements were performed using conventional ELISA rather than ultrasensitive digital immunoassays (e.g., Simoa), which may affect analytical sensitivity, dynamic range and inter-assay variability. Although all samples were analysed under identical experimental conditions within the same assay batch, future longitudinal studies would benefit from validation using automated ultrasensitive assay platforms. Furthermore, neurological phenotyping was relatively limited and primarily based on the PND score. Future studies would benefit from incorporation of more detailed clinical, electrophysiological, and quantitative sensory assessments.

## Conclusions

In conclusion, serum neurofilament light chain (sNfL) is a sensitive and dynamic biomarker of axonal degeneration in hereditary transthyretin amyloidosis. Its elevation across disease stages and correlation with neuropathy severity suggest that sNfL could facilitate objective assessement of disease severity and progression.

By contrast, sGFAP and sPRPH did not exhibit significant group differences, likely reflecting their tissue specificity and release kinetics. While sNfL holds clear promise for clinical application, further longitudinal studies with larger cohorts are required to establish prognostic cut-offs and to evaluate its responsiveness to emerging disease-modifying therapies.

## Data Availability

Data relevant to the current study can be made available (after ethics and confidentiality review) upon reasonable request from the corresponding author.
